# Supplementary material for: How Little Do We Know about HIV and STIs Prevention? Results from a Web-Based Survey among the General Population
Source: Healthcare (Basel). 2022 Jun 8;10(6):1059. doi: 10.3390/healthcare10061059 (PMC9222353; doi:10.3390/healthcare10061059)
Supplement: Supplementary file 1 [file healthcare-10-01059-s001.zip › healthcare-1753884-supplementary.pdf]

the level of awareness about HIV and STIs

1. **How old are you? (Open question)**
2. **Gender**
   Male
   Female
   Transgender female
   Transgender male
   Other
3. **Region of origin** (All the Italian regions were listed as options)
4. **Educational level:**
   None
   Primary school
   Middle school
   Secondary school
   University degree or higher
   I prefer not to answer
5. **Occupation**:
   Unemployed
   Retired
   Self-employed
   Employee
   Temporary job
   Student
   Housewife
   Health worker/health profession student
   I prefer not to answer
6. **What is HIV? (only one corrected answer)**
   A virus
   A bacterium
   A fungi
   I do not know
   It does not exist
   Other
7. **How is it possible to transmit HIV?**
   Through kisses
   Through blood
   Through sexual intercourse
   During labour
   I do not know
8. **Through which of the following is HIV transmitted?**
   Saliva
   Blood
   Vaginal discharge
   Sperm
   Sweat
   Urine
   I do not know
9. **How is it possible to recognize a person living with HIV?**

It is not possible
From skin patches
He/She would be extremely thin
He/She would look like an ill person
I do not know

10. **Is it dangerous to live with a person living with HIV?**
Not at all
Yes, by sharing services
Yes, by drinking from the same glass
Yes, by sharing kisses and hugs
I do not know
11. **In your opinion, if a person living with HIV timely assumes his/her treatment and has an undetectable viral load, can He/She transmit the virus?**
(Yes or No answer)
12. **What is the age at higher risk of contracting a sexually transmitted infection? (only one corrected answer)**
15-25
30-40
40-50
>50
I do not know
13. **Does contraceptive pill protect from sexually transmitted infections? (only one corrected answer)**
No, never
Yes, always
It depends on the woman's age
I do not know
14. **Which are the microorganism responsible for sexually transmitted infections?**
Viruses
Bacteria
Protozoa
I do not know
15. **How is it possible to acquire sexually transmitted infections?**
Through saliva
Through sperm
Through vaginal discharge
Through cough and sputum
I do not know
16. **How is it possible to prevent sexually transmitted infections and HIV transmission?**
Using condoms during the whole and all sexual intercourses
With proper intimate cleansing after each sexual intercourse
By assuming contraceptive pills
By practicing interrupted coitum
I do not know
17. **In which kind of sexual intercourse, do you have the highest chance of HIV and sexually transmitted infection transmission?**
Receptive intercourse
Penetrative intercourse
Both
There is not risk of any kind

I do not know

18. **Have you ever had sexual intercourse in your life?**
    (Yes, No, I prefer not to answer)
19. **During an occasional sexual intercourse, how often do you use condoms?**
    Always
    Almost always
    Sometimes
    Never
    I prefer not to answer
20. **Have you ever heard of U=U campaign? (Yes or No answer)**
21. **Do you think taking part to this survey could have been the chance to learn more about this topic?** (Yes or No answer)
